# Rotating Magnetic Field Mitigates Ankylosing Spondylitis Targeting Osteocytes and Chondrocytes via Ameliorating Immune Dysfunctions

**DOI:** 10.3390/cells12070972

**Published:** 2023-03-23

**Authors:** Yu Han, Hua Yang, Zhongke Hua, Shenglan Nie, Shuling Xu, Cai Zhou, Fengyi Chen, Mengqing Li, Qinyao Yu, Yang Sun, Yunpeng Wei, Xiaomei Wang

**Affiliations:** 1 Magnetobiology Group, Department of Physiology, Shenzhen University Health Science Center, Xili Campus of Shenzhen University, Shenzhen 518055, China; 2 State Key Laboratory of Pharmaceutical Biotechnology, Department of Biotechnology and Pharmaceutical Sciences, School of Life Sciences, Nanjing University, Nanjing 210023, China

**Keywords:** ankylosing spondylitis, rotating magnetic field, CD4-CKO mice, cartilage tissues, inflammation

## Abstract

Ankylosing spondylitis (AS) is clinically characterized by bone fusion that is induced by the pathological formation of extra bone. Unfortunately, the fundamental mechanism and related therapies remain unclear. The loss of SHP-2 (encoded by *Ptpn11*) in CD4-Cre;*Ptpn11*^f/f^ mice resulted in the induction of AS-like pathological characteristics, including spontaneous cartilage and bone lesions, kyphosis, and arthritis. Hence, this mouse was utilized as an AS model in this study. As one of the basic physical fields, the magnetic field (MF) has been proven to be an effective treatment method for articular cartilage degeneration. In this study, the effects of a rotating magnetic field (RMF; 0.2 T, 4 Hz) on an AS-like mouse model were investigated. The RMF treatment (2 h/d, 0.2 T, 4 Hz) was performed on AS mice from two months after birth until the day before sampling. The murine specimens were subjected to transcriptomics, immunomics, and metabolomics analyses, combined with molecular and pathological experiments. The results demonstrated that the mitigation of inflammatory deterioration resulted in an increase in functional osteogenesis and a decrease in dysfunctional osteolysis due to the maintenance of bone homeostasis via the RANKL/RANK/OPG signaling pathway. Additionally, by regulating the ratio of CD4+ and CD8+ T-cells, RMF treatment rebalanced the immune microenvironment in skeletal tissue. It has been observed that RMF interventions have the potential to alleviate AS, including by decreasing pathogenicity and preventing disease initiation. Consequently, RMF, as a moderately physical therapeutic strategy, could be considered to alleviate the degradation of cartilage and bone tissue in AS and as a potential option to halt the progression of AS.

## 1. Introduction

Ankylosing spondylitis (AS) is an autoimmune disease that targets the central nervous system (CNS), particularly the axial region [1]. Human leukocyte antigen-B27 (HLA-B27) has been demonstrated to have a considerable relationship with this disorder (heritability > 90%) [2,3]. Pathological fresh bone generation is a prominent hallmark of AS, which differs considerably from the clinical symptoms of rheumatoid arthritis (RA) aside from inflammation of the backbone and rheumatic impairment [4]. Osteogenesis emerges in two stages: intramembranous and endochondral ossification [5,6]. The growth plate comprises chondrocytes, which migrate directly through a series of narrowly regulated developmental stages, from round cells in the resting zone to columnar cells in the proliferative zone, and ultimately to elongated cells in the pre-hypertrophic and hypertrophic zones, where ossification arises [7]. Chondrocytes emerge from a lineage of cells that are overwhelmingly less multipotent, such as mesenchymal stem cells (MSCs), skeletal system stem cells (SSCs), bone chondrocyte stromal cell precursor cells (BCSPs), pre-chondrocyte cells (PCPs), resting chondrocytes, and pre-hypertrophic/hypertrophic chondrocytes located within end bones and growth plates [7,8].

Arthrosclerosis, spinal ankylosis, and even lifelong paralysis develop in AS patients once osteophytes have thoroughly replaced the cavity of the afflicted joint [9]. Nonsteroidal anti-inflammatory drugs (NSAIDs), tumor necrosis factor-α (TNF-α) antibodies, and interleukin-17A (IL-17A) inhibitors could efficiently suppress inflammation in aggressive AS. Nevertheless, none of these medications appreciably retarded the radiographic deterioration of AS [10,11,12]. However, experts have been hitherto unable to elucidate the mechanisms behind malignant new bone synthesis. For nascent bone development in spinal syndesmophytes, MRI data suggested that the regions with pre-existing inflammation were more likely to exhibit new bone synthesis than those without underlying inflammation [13]. The generation of osteoinductive molecules and the construction of abnormal fresh bone were also stimulated by inflammation [14,15]. Fresh bone construction is not only driven by inflammation, as most additional vertebral syndesmophytes emerge at places with no indications of recent inflammation [16,17]. Endochondral ossification, the mechanism by which periosteal cells differentiate into chondroblasts and osteoblasts, generates the osteophytes [18]. Recently, it has been demonstrated that the ligaments of individuals with early AS exhibit chondrocyte differentiation, which culminates in cartilage fabrication and subsequent myotenositis [19]. Alternatively, osteoporosis is ubiquitous in AS, with an incidence of 9.5–40% [20,21]. In AS, an overabundance of osteoclasts was observed on the bone surfaces of calcified cartilage and subchondral bone marrow [19,22]. According to the aforementioned studies, both chondrogenesis and osteoclastogenesis play a role in the development of AS.

Src homology region two-domain-containing phosphatase-2 (SHP-2), a commonly expressed protein that is encoded by protein tyrosine phosphatase nonreceptor type 11 (*Ptpn11*), acts as a regulator of cell growth, proliferation, and differentiation [23]. The deficiency of SHP2 results in prenatal fatalities [24]. Interestingly, T-cells stimulated by SHP2 are inhibited by the programmed death receptor 1 (PD-1), which is triggered by extracellular signal-related kinase (ERK) downstream of growth factor and cytokine receptors [25,26]. Researchers have indicated that SHP-2 is critical for the formation and function of embryonic hematopoietic stem cells (HSCs) and MSCs [27]. Various forms of carcinoma, such as leukemia, esophageal, and oropharyngeal cancers, have been connected with the *Ptpn11* gene mutations, which cause Noonan and Leopard syndromes [28,29]. Metachondromatosis, a unique condition resulting in malignancies of cartilage tissues, is induced by loss-of-function mutations of SHP-2 [30,31]. Since SHP-2 acts as both an agonist and an antagonist in various signaling pathways, it is crucial to have a better knowledge of SHP-2 signaling dependent on cell subtypes and pathways [32]. Exostoses, dwarfism, and restricted bone calcification can all be triggered by the conditional deletion of SHP2 in various chondrocyte subsets [31,33,34,35]. In chondrocytes from mice, conditional deletion of *Ptpn11* impairs terminal differentiation and growth plate morphology [34]. Furthermore, a conditional knockout of Ptnp11 in chondrocytes indicated that SHP-2 plays a vital role in the osteogenic fate of hypertrophic chondrocytes [36] and in the modulation of SOX9 activity [37]. It has also been discovered that, although SHP-2 is expressed on all mature T-cells in the thymus, it induces skeletal abnormalities in mice [38]. The dysfunction of SHP-2 downstream elements, including Ras guanine exchange factors (RasGEFs) SOS-1 and -2 [39], ERK-1, and -2 in CD4^+^ cells, also aggravates skeletal lesions in animals defective in these downstream constituent parts [40]. When SHP2 was deprived, metachondromatosis was induced, as were the synthesis of Indian hedgehog (Ihh) and the stimulation of parathyroid hormone-related protein (Pthrp), whereas these effects were alleviated after the utilization of the smoothened (Smo) inhibitor [31]. Thus, these investigations elucidate a probable relationship between SHP2 and chondrocyte formation, which remains to be ascertained.

Serendipitously, in this study, a spontaneous AS-like degenerative disease was observed in senior CD4-Cre;*Ptpn11*^f/f^ mice that exhibit signs of kyphosis, scoliosis, arthritis, and bone fusion in the axial joints. Lineage tracing and functional investigation indicated that a SHP2 deficit in pre- and hypertrophic chondrocytes impairs epiphyseal fusion (cartilaginous endplate and growth plate). Hence, the CD4-Cre;*Ptpn11*^f/f^ mouse is a valuable mouse model for AS.

Magnetic field (MF) is progressively utilized as a therapeutic technique in basic academic investigations. Additionally, MF treatment is a convenient and non-invasive approach to addressing a wide range of medical conditions, including injuries, pain, and inflammation, as well as a wide range of other ailments [41]. Furthermore, the magnetite-based electron eddy theory provides a robust analytical and empirical basis for magnetic treatments [42]. In previous studies, multiple endogenous mechanisms regulating inflammation and healing have been demonstrated to explain the changes in animal activities after MF exposure [43]. Moreover, animals and plants are able to perceive the amplitude, frequency, strength, and orientation of MF through specific magnetic induction sensors [44,45]. Numerous efforts have been made to explore the signaling pathways involved in magnetic field perception.

Generally, MFs can be divided into the static and variable magnetic fields. The intensity and direction of the static magnetic field do not change, while the intensity and direction of the variable magnetic field shift with time. As one of the common variable magnetic fields, a rotating magnetic field (RMF) is a magnetic field that changes the direction of the magnetic field at a constant angular rate and period, which is typically generated by the circling motion of permanent magnets driven by a motor [46]. Until now, RMF has rarely been used in biomedical research, but various bioeffects of RMF have been discovered, including adjusting cell differentiation [47], regulating cytokine production [48], increasing bone mineral density [49], ameliorating oxidative stress in the heart [50,51], and improving the heat distribution of the body surface [52]. Currently, there is less data on the impact of MFs on AS disease in the available literature. Furthermore, most studies on orthopedic disorders, both in vivo and in vitro, have concentrated on the static magnetic field without considering the biological effects of RMF.

In this study, the effects of RMF on the attenuation of immune disorders, cartilage lesions, and spinal cord demyelination in AS mice were reported for the first time.

## 2. Materials and Methods

### 2.1. Mice and Experimental Design

CD4-Cre;*Ptpn11*^f/f^ (CD4-CKO) mice were produced by crossing *Ptpn11*^f/f^ mice with CD4-Cre transgenic mice [53]. All these mice were of C57BL/6 background and were obtained from GemPharmatech Co., Ltd. (Nanjing, China). The mice were placed in plastic cages at a temperature of 25 ± 2 °C with a 12-h light/dark cycle and free access to forage and water. The mice were then assigned to the following four groups (*n* = 6 each): (1) sham group (wild type), (2) RMF group (wild type + RMF), (3) AS group (CD4-CKO), and (4) AS + RMF group (CD4-CKO + RMF). For 6 months after modeling, we employed the RMF to treat AS mice.

All mice were bred in a dedicated pathogen-free animal house at Shenzhen University. The Animal Ethical and Welfare Committee of Shenzhen University examined and confirmed the animal utilization and experimental protocols in conformity with the guidelines established by the Animal Ethical and Welfare Committee. Every procedure was performed to minimize animal suffering and to limit the number of animals utilized.

### 2.2. Scoring Severity of Bone Disease and Athletic Ability

After modeling and RMF treatment of mice, the mice were scored for motor ability and disease progression in the four groups (Table 1 and Table 2) [54].

### 2.3. Rotating Magnetic Field

**Figure 1:**
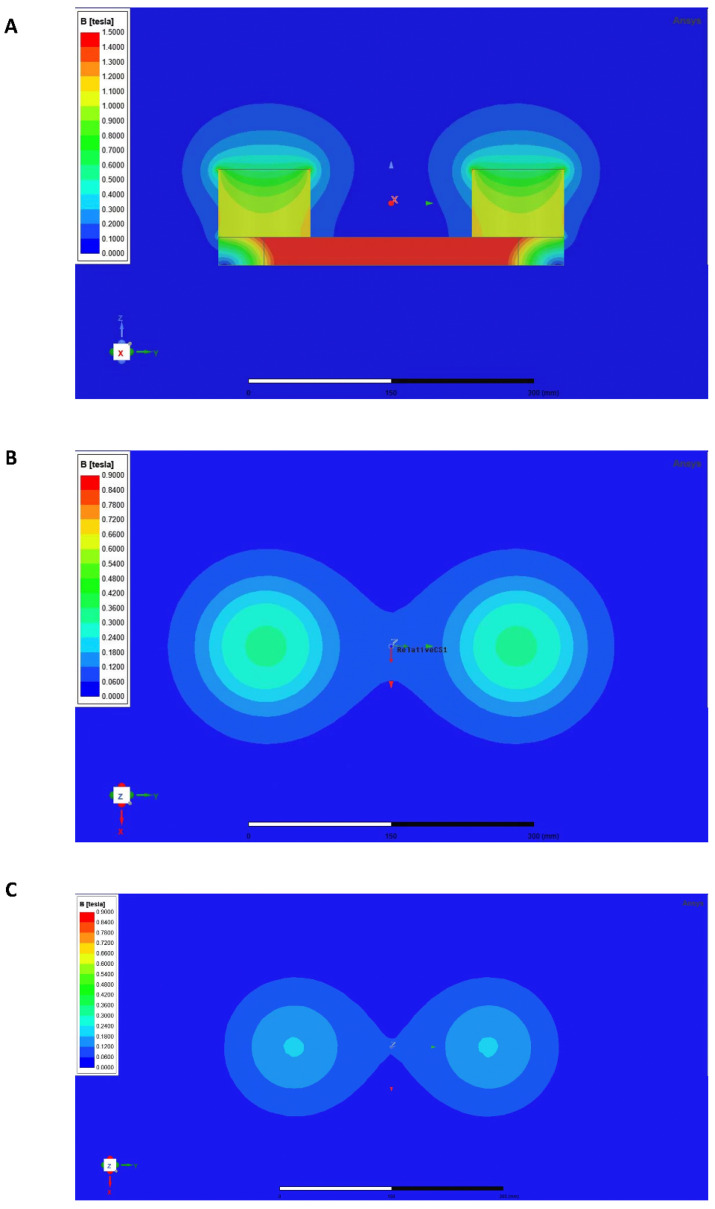
The RMF exposure apparatus. (**A**) The physical parameters of the treatment apparatus, which consists of two antiparallel cylindrical NdFeB (neodymium iron boron) permanent magnets (cross-section drawn). (**B**) The simulation of magnetic field intensity on the murine podalic exposure plane (vertical view). (**C**) The simulation of magnetic field intensity on murine dorsal exposure flat (vertical view).

Previously published studies established the entire fabrication of the RMF exposure apparatus [49,55]. The RMF apparatus was constructed using two antiparallel Neodymium Iron Boron (NdFeB) permanent magnets (Figure 1A) manufactured with the Rotating Magnetic Bed System (RMBS, designed by Prof. Xiaoyun Zhang, Shenzhen University, Shenzhen, China). The diameter of each magnet is 98 mm, the height is 72 mm, and the center distance of the two magnets is 270 mm. Individually Ventilated Cages (IVC) were custom-made for the RMF and installed in the device’s core. The RMF device’s intensity was assessed utilizing a three-axis fluxgate magnetometer (GMW Associates, Redwood, CA, USA) and a Hall magnetometer (ETM-13-Achsen, Geneva, Switzerland). Following our previous investigations, RMF exposure was considered for 2 h per day in an RMF with an average amplitude of 0.2 T and a rotating frequency of 4 Hz [49,55]. The RMF treatment was performed on mice in each group from the second month after birth to the day before euthanasia.

When exposed to the RMF, the murine podalic flat is 250 mm away from the top of the magnet, and the dorsal flat is 450 mm away from the top of the magnet. A magnetic field with variable intensity was generated above the device by the rotation of two parallel cylindrical NdFeB permanent magnets with opposite magnetization orientations fixed to an iron plate. Hence, we simulated the intensity distribution of magnetic fields at the murine podalic flat and dorsal flat using ANSYS Maxwell 2020R1 (Figure 1B,C).

### 2.4. Locomotor Behavioral Test

Following the modeling, mice have been separately accommodated in Pheno Typer 3000 cages (Noldus, Wageningen, Netherlands). After the mice became accustomed to their new habitat, their movement within the cage was monitored using animal video tracking software Ethovision XT (Noldus, Wageningen, Netherlands). After that, a mouse behavioral trajectory image was generated via the Ethovision XT V14 processing system (Noldus, Wageningen, Netherlands), with the entire moving distance and the average motion speed of mice measured.

Rota-rod training was utilized in this study to assess murine physical capacity. The diameter of the rotary rod (ZB-200, Shanghai, China) is 8 cm, comprising 5 channels, with each channel being 11 cm wide and having a ribbed surface. Computers were utilized to regulate rotational velocity (1 cycle/s). The mice acclimated to the spinning rod within 10 s and continued walking for 5 min. The mice were scored according to the length of time they continued to crawl on the rotating bar within the prescribed time (Table 3).

Afterward, four groups of mice were forced to run on the experimental animal running platform (ZB-200, Shanghai, China) (the racetrack was sloped at 11 degrees, and the pace was maintained at 10 m/min). An electrical stimulation (50 V) on the tails of mice was designed to motivate them to move forward. This sufficiently drove the mice to run until they had been on the treadmill for 5 min. Experimentally, the mice were scored according to the number of electric shocks they received during the prescribed running time (Table 4).

### 2.5. Radiography Analysis

Mice were inspected with a fixation X-ray system (IVIS Lumina XR, PerkinElmer, Waltham, MA, USA), and the femur’s bone mineral density (BMD) was determined by a dual-energy X-ray absorptiometry system. Scans of uncalcified specimens were carried out using X-ray microtomography (micro-CT, Quantum GX2, PerkinElmer, Waltham, MA, USA) at 50 kV, 80 µA, 0.5-mm aluminum filtering, and 9-µm isotropic resolution. Each specimen was scanned for a total of 4 min. Using Analyze 14.0 software (USA), the bone tissue 1 mm from the end of the distal femoral growth plate with a layer thickness of 2 mm was selected as the region of interest (ROI) for 3D reconstruction of bone trabeculae. The bone tissue 3 mm from the distal femoral growth plate and extending 1 mm in thickness toward the backbone was selected as the ROI for cortical bone reconstruction. The image information was extracted with a minimum threshold of 150, and several parameters were quantitatively analyzed with the Analyze 14.0 software, including the BMD, cortex, intra-trabecular tissue, and trabeculae.

### 2.6. Skeletal Preparations

The bones of mice were preserved in 10% formaldehyde solution for 48 h, decalcified in 10% ethylenediamine tetraacetic acid (pH 7.4) for 21 days, and embedded in paraffin with an optimal cutting temperature substance (Leica, Wetzlar, Germany). Afterwards, the cartilage samples were sequentially dehydrated in ethanol (70%, 2 h × 2; 95%, 2 h × 2; 100%, 2 h × 2) and subsequently washed in xylene (2 h × 2) before being embedded in paraffin (Leica, Wetzlar, Germany). Specimens were sliced into 5–7 μm segments and converted to adhesive glass slides (Superfrost Plus Adhesion Slide, Thermo Fisher Scientific, Waltham, MA, USA) via a microtome (Leica, Wetzlar, Germany). The segment slides were deparaffinized in xylene (15 min × 2) and serial ethanol (100%, 5 min × 2; 95%, 5 min × 2; 80%, 5 min; 70%, 5 min). Ultimately, the slides were rinsed and maintained in cold double-distilled water (DDW) until they were hydrated.

### 2.7. Histological Staining

The paraffin specimens were segmented into 5 μm of thickness. Proteoglycans in the cartilage were stained with Safranin O/Fast Green. After rehydrating the skeletal section slides with distilled water, the tissue slices were incubated with Weigert’s iron hematoxylin (Solarbio, Beijing, China), safranin O (2% *w*/*v*), and fast green solution (0.05% *w*/*v*). The slides were dehydrated in 95% ethyl alcohol and absolute ethyl alcohol, respectively, and cleared with xylene after 1 min of flushing. Resinous mounting material was applied to mount the slide. Microscopy was performed to gain the images (Olympus, Tokyo, Japan). The extent of cartilage deterioration was graded on a scale of 0–6 according to the OARSI (Osteoarthritis Research Society International) scoring system [56], and images were obtained using the CellSens Standard software (Olympus, Tokyo, Japan).

All specimens were stained with H&E (hematoxylin and eosin, ORIGENE, Beijing, China). To separate tints, HCl-EtOH (1% *v*/*v*) and ammonia-H_2_O (0.2% *v*/*v*) were utilized. After dehydration with alcohol and xylene, the resin was applied to mount tissue slice samples. Finally, the photos were captured using microscopy (Olympus, Tokyo, Japan).

### 2.8. Immunohistochemistry

After hydration, sample slices were incubated with 3% H_2_O_2_ for 15 min. The tissue slices were then blocked with 5% goat serum (Proteintech, Rosemount, PA, USA) at 37 °C for 30 min. Sections were incubated with OPG (1:200, Santa Cruz Biotechnology, Dallas, TX, USA) and RANK (1:200, Santa Cruz Biotechnology, Dallas, TX, USA) antibodies at 4 °C overnight. After 3 washes with phosphate-buffered saline containing 0.1% tween-20 (PBST), the tissue slices were incubated with horseradish peroxidase (HRP)-conjugated goat anti-mouse/rabbit secondary antibody (ORIGENE, Beijing, China) at 37 °C for 30 min. Then, a diaminobenzidine (DAB) color development kit (ORIGENE, Beijing, China) was used to generate the colors. After dehydration with alcohol and xylene, the resin was applied to mount tissue slice samples. The images were obtained using microscopy (Olympus, Tokyo, Japan).

### 2.9. Immunofluorescence Staining

After dewaxing and hydrating the paraffin-embedded slices (5 μm), the antigen was recovered with sodium citrate (Beyotime Biotechnology, Nanjing, China). After blocking the sections with 5% goat serum (Proteintech, Rosemount, PA, USA), tissue slices were treated with the anti-CD3 (1:100, Cell Signaling Technology, Danvers, MA, USA) antibody at 4 °C overnight. Before immunofluorescence dye, secondary antibodies: goat anti-rabbit IgG2a coupled to Alexa Fluor 488 (1:200, Invitrogen, Waltham, MA, USA) were applied to probe the slices. The sections were photographed using a fluorescence microscope (Olympus, Tokyo, Japan) after being counterstained with DAPI (Abcam, Cambridge, UK).

### 2.10. Enzyme-Linked Immunosorbent Assay and Cytometric Bead Array

The serum was extracted after centrifuging the blood at 1000× *g* at 4 °C for 15 min. Serum aliquots were obtained and preserved at −20 °C until examination. Serum cytokines IL-5, IL-17A, IL-22, IL-23, IL-28, and IFN-γ were quantified using a cytometric bead array (CBA) in accordance with the murine cytokine kit (BD, Franklin, TN, USA). To further verify the result, the serum cytokine concentrations, including IL-17A, IL-22, and IL-23, were assessed via an enzyme-linked immunosorbent assay (ELISA) kit (Invitrogen, Waltham, MA, USA) according to the manufacturer’s guidelines.

### 2.11. Murine Lymphocyte Isolation

Isoflurane medication was applied to euthanize the mice at the stated ages. Before harvesting the organs, a cardiac puncture was administered to obtain sufficient blood samples for further investigation. Then, the spleens were separated, and lymphocytes were extracted via Murine Lymphocyte Cell Separation Media (Dakewe Biotech Co., Ltd., Beijing, China), which were purified with a 40–70% discontinuous percoll gradient (Dakewe Biotech Co., Ltd., Beijing, China) [57].

### 2.12. Flow Cytometry Analysis and Antibodies

Mononuclear cells were cultivated in RPMI 1640 media (Corning, New York, NY, USA) with 10% FBS (Gibico, Invitrogen, Waltham, MA, USA) and 1% penicillin-streptomycin combination (PS, Hyclone, Logan, UT, USA) at 37 °C in a thermal incubator with 5% CO_2_ for 5 h. Cell surface markers, including fluorochrome-conjugated CD4 and CD8 antibodies (BD, Franklin, TN, USA), were applied to label the lymphocytes. The cells were softly mixed with the markers and incubated at 4 °C in the dark for 30 min. A total of 50,000 fluorescence events were aggregated. Negative controls were unlabeled cells.

### 2.13. Immunoblotting

The immunoblot experiment was performed as previously reported [58]. Total proteins were extracted from murine specimens using the Ripa buffer (Beyotime, Shanghai, China) with a protease inhibitor cocktail (MCE, Monmouth junction, NJ, USA). The protein concentration was determined using a Pierce^TM^ BCA Protein Assay Kit (Thermo Fisher Scientific, Waltham, MA, USA). Using SDS–polyacrylamide gel electrophoresis, proteins (20 μg) in lysis buffer were isolated and electrophoretically deposited onto polyvinylidene difluoride membranes (Millipore, Billerica, MA, USA). The membranes were stained with antibodies against OPG (1:1000, Santa Cruz Biotechnology, Dallas, TX, USA) and RANK (1:1000, Santa Cruz Biotechnology, Dallas, TX, USA) at 4 °C overnight and then addressed with a secondary antibody conjugated to horseradish peroxidase (1:5000, Cell Signaling Technology, Danvers, MA, USA). The LumiGLO chemiluminescent substrate technique was utilized for detection.

### 2.14. Total RNA Extraction and RNA Sequencing

The euthanasia was performed after the mice were placed into slumber using isoflurane. Afterwards, RNA was extracted from the specimens with a total RNA extraction kit, which was subsequently processed following the manufacturer’s instructions (Promega, Madison, WI, USA). After isolation, the RNA pellets were resuspended in 1 mL of 75% ethyl alcohol. Centrifuge the pellet at 7500 rpm at 4 °C for 5 min to obtain purer RNA samples. Then, the RNA pellets were placed in a biosafety cabinet, allowing them to dry completely in the air. For further investigation, the RNA pellet was diluted in 25–100 μL of RNase-free water.

The data were indexed, compiled, and sequenced (BGI Genomics, BGI-Shenzhen, Shenzhen, China) using the Illumina HiSeq. Illumina’s BCL2FASTQ software and a bespoke Python demultiplexing tool were utilized for base-calling and demultiplexing. In order to evaluate the performance of each gene, we plotted the residual standard deviation of each gene against its average log count and depicted a trend line that matched the residuals fairly closely. After performing the differential expression analysis, Benjamini-Hochberg adjusted *p*-values less than or equal to 0.05 were selected to determine treatment differences. For each contrast retrieved through Limma, global variations in well-known Gene Ontology (GO) keywords and the Kyoto Encyclopedia of Genes and Genomes (KEGG) were detected using the R/Bioconductor program GAGE8. Heatmap3 was utilized to depict GO item data with a Benjamini-Hochberg corrected P-value of less than or equal to 0.05. Furthermore, *p*-values less than or equal to 0.05 were used to generate annotated graphs for the disturbed KEGG pathway, where the estimated log 2-fold variation of genes within the term was considerably perturbed in a single direction versus the background or in either direction relative to other genes within the phrase.

### 2.15. Quantitative Polymerase Chain Reaction

When tissues were harvested, total RNA was extracted with Trizol reagent (Invitrogen, Waltham, MA, USA). The reverse transcription reaction of RNA was performed using the Evo M-MLV RT Mix Kit with gDNA Clean for qPCR (AG, Guangzhou, China), and the reaction was then quantified via a Bio-Rad T100^TM^ PCR apparatus (Bio-Rad, Hercules, CA, USA). From reverse transcription to amplification reactions, we produced particular primers that span the back spliced junction and executed qPCR with the SYBR^®^Green Premix Pro TaqHS qPCR Kit (Rox Plus) (AG, Guangzhou, China). Every reaction was performed four times, and the results were standardized to the housekeeping gene β-actin (Table 5). The 2^−ΔΔCt^ methodology was applied to quantify the relative mRNA expression levels.

### 2.16. Metabolomics Detection

The serum was obtained via a 15-min, 4 °C centrifugation of the blood at 1000× *g*. Metabolomic analysis was performed on serum specimens from each sample. In order to detect metabolites in murine serum, the tandem mass spectrometry (MS/MS, Applied Biosystems QTRAP) system and ultra-performance liquid chromatography (UPLC, Shim-pack UFLC SHIMADZU CBM30A) were applied (Metware Co., Ltd., Wuhan, China). Additionally, principal component analysis (PCA) was performed to illustrate intragroup variance and identify probable outliers. The resulting matrix was also implemented concurrently with PLS-DA and OPLS-DA analyses to support profile visualization and differentiation for the corresponding datasets. A standard criterion was used to pick important variables: *p*  <  0.05.

### 2.17. Statistical Analysis

Prism 8.0 (Graph-Pad Software, Inc., San Diego, CA, USA) was applied for statistical analysis. An unpaired, two-tailed Student’s *t*-test was utilized to compare two respective groups. The variance of multiple groups was compared using a one-way analysis of variance (one-way ANOVA). The error bars represent the standard error of the mean, and * *p* < 0.05, ** *p* < 0.01, ns, no significance.

## 3. Results

### 3.1. RMF Alleviates Age-Related AS-like Bone Disease Caused by Ptpn11 Deletion in CD4-Expressing Cells

**Figure 2:**
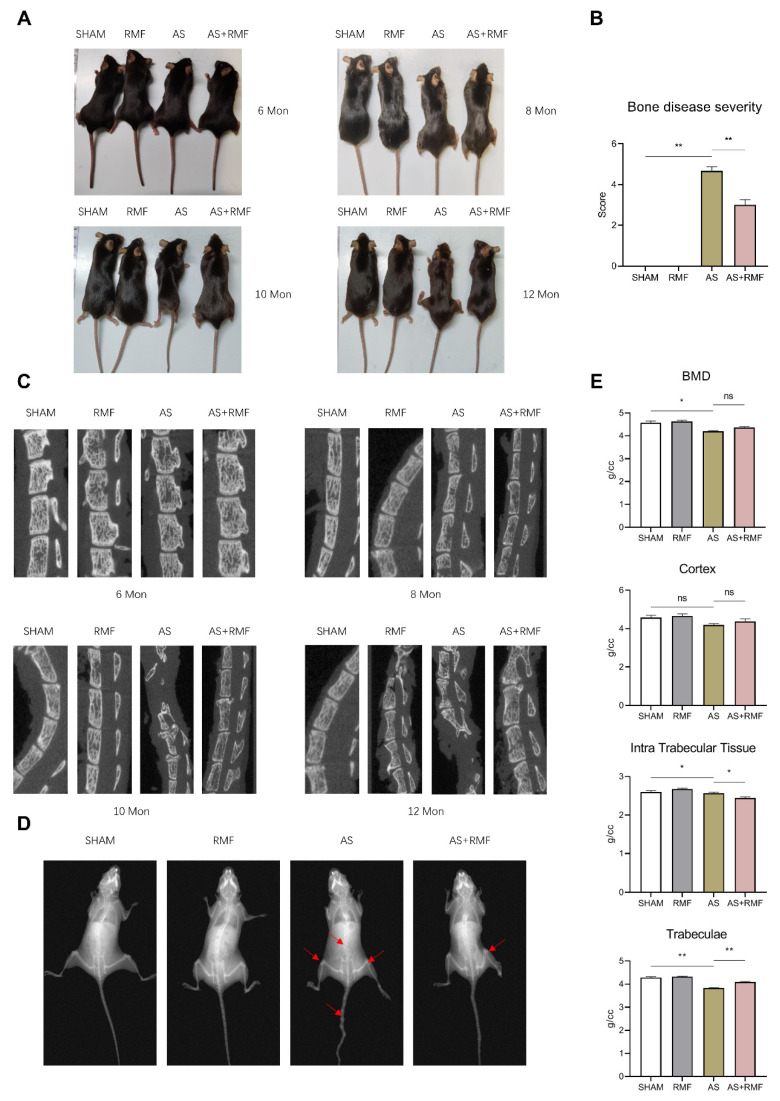
RMF treatment alleviates cartilage degradation and bone fusion in age-related AS-like CD4-CKO mice. (**A**) Gross photographs of female mice in four groups at 6, 8, 10, and 12 months of age (*n* = 6). (**B**) Pathological score (*n* = 6) of mice. (**C**) Micro-CT radiographs display the spine’s bone structure and intervertebral discs (*n* = 6). (**D**) X-ray films of four groups of mice at 12 months old. Red arrows exhibit joint ankylosis and kyphoscoliosis in 12-month-old AS and AS + RMF mice (*n* = 6). (**E**) Femoral bone mineral density (BMD, *n* = 6) of cortical and subchondral bone in four groups of 12-month-old AS mice. (**B**,**E**) Data are presented as the mean ± SEM, * *p* < 0.05, ** *p* < 0.01, ns, no significance, by one-way ANOVA.

The CD4-Cre;*Ptpn11*^f/f^ (CD4-CKO) mice were produced by crossing *Ptpn11*^f/f^ (CD4-Ctrl) mice with mice expressing Cre under the control of the endogenous CD4 promoter. The bone pathology in CD4-CKO mice was comparable to the radiological abnormalities observed in humans with AS, which is characterized by structural deterioration and osteointegration in axial joints such as the spine, sacroiliac, and knee joints. It was observed that CD4-CKO mice spontaneously developed bone disease 6 months after birth, which originated with an enlarged pelvic incidence angle and knee stiffness, and progressed to hip and knee kyphosis and ankylosis, respectively. In contrast, these pathological alterations were ameliorated following RMF treatment (Figure 2A,B). Structural degradation and bone fusion resulted in persistent impairment, manifested by spine deformity, reduced motor capacity, and weight loss observed in CD4-CKO-AS mice from 6 months of age. Under RMF intervention, the symptoms described above were modest without evident spinal deterioration, and impairment was largely alleviated, particularly bone fusion and osteophytes involving the spine, wrist, sacroiliac joint, as well as hip and knee joints (Figure 2A).

Micro-CT analysis demonstrated that RMF remitted minor bone deformation and osteophytes in the spines of adult AS mice aged 6, 8, 10, and 12 months, accompanied by decreased osteophytes, bony fusions, and ankylosis in the spines of elderly AS mice (Figure 2C). AS mice, in particular, exhibited significant intervertebral cartilage hyperplasia at 10 and 12 months of age, compressing the surrounding bone and causing severe spinal curvature and distortion. Interestingly, RMF alleviated AS-like mice’s exorbitant intervertebral cartilage hyperplasia at 10 and 12 months, which squeezed the adjacent bone and led to extensive spinal curvatures and deformations (Figure 2C). However, AS mice treated with RMF consistently developed moderate cartilage hyperplasia.

RMF treatment was observed to reduce arthrosis, articular stiffness, and decrease the substantial loss of compact and subchondral bone (red arrow) in 12-month-old AS mice using X-ray imaging. The femoral bone mineral density (BMD) was considerably reduced in AS mice compared to the AS-RMF littermates, followed by a substantial decline in cortex and trabeculae but not in intra-trabecular tissue (Figure 2D,E).

### 3.2. RMF Mitigates Athletic Injury and Age-Related Articular Deterioration in AS Mice

**Figure 3:**
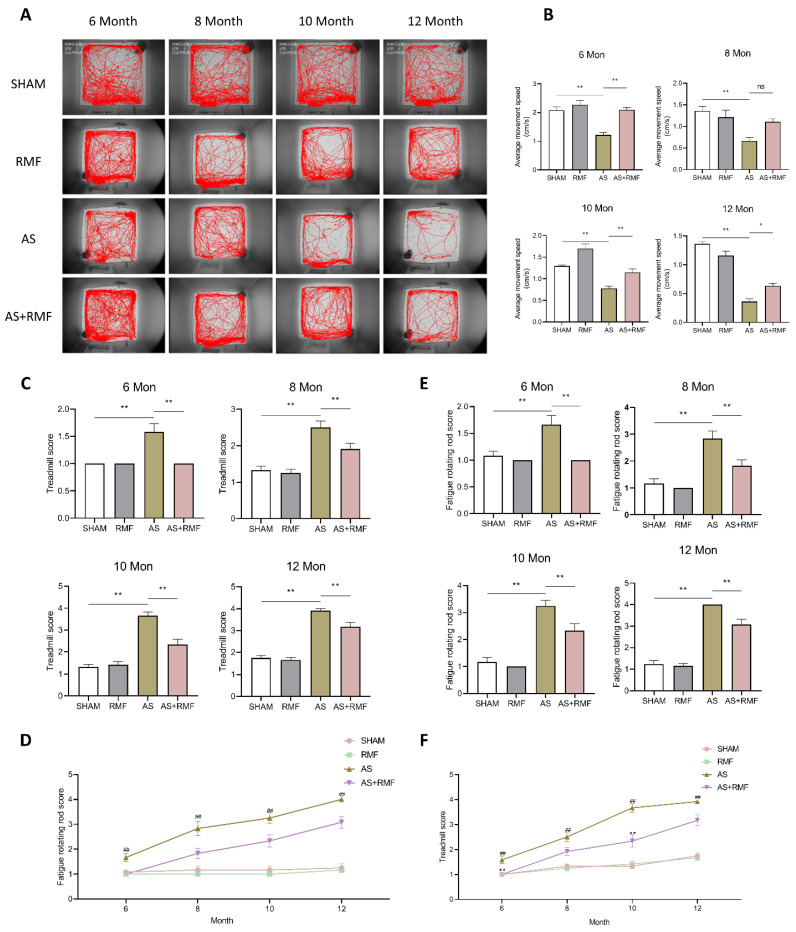
RMF mitigates athletic ability and age-related articular deterioration in AS mice. (**A**) The behavior trace images were analyzed via an open field test (*n* = 6). (**B**) Average locomotor speed of mice among four groups at 6, 8, 10, and 12 months old (*n* = 6). (**C**,**D**) The movement score of mice at 6, 8, 10, and 12 months old in the treadmill experiment (n = 6). (**E**,**F**) The movement score of mice at 6, 8, 10, and 12 months old in the fatigue rotating rod experiment (*n* = 6). (**B**,**C**,**E**) Figures are presented in the form of the mean ± SEM, * *p* < 0.05, ** *p* < 0.01, ns, no significance, by one-way ANOVA. (**D**,**F**) Figures are presented in the form of the mean ± SEM, ** *p*: Sham vs. AS, ## *p*: AS vs. AS + RMF.

An open-field experiment was conducted to examine the psychomotor capabilities of mice. AS mice exhibited a decline in function at 6 months of age, followed by an accelerated reduction in activity and an almost complete loss of non-essential exercises other than drinking and foraging at 12 months of age (Figure 3A). Additionally, the AS + RMF and AS groups displayed a substantial disparity in activity measured from 6 months of age onward (Figure 3A), and successive symptom progression in the AS + RMF group was alleviated. At 12 months of age, the mice in the AS + RMF group retained their capacity to perform basic exercises (Figure 3A,B). Nevertheless, decreased activity does not inevitably imply decreased athletic ability. Subsequently, mouse treadmill and fatigue rotation rod experiments were conducted to assess murine locomotor capability. The mice were compelled to run on a sloping treadmill, and their exercise capacity was graded into 4 categories depending on the frequency of times they were shocked in the tail for struggling to keep up with the racing exercise. Activity damage was more severe in AS mice, whereas it was less severe in AS mice treated with RMF (Figure 3C,D). Simultaneously, similar trends were discovered in the murine fatigue rod rotation test (Figure 3E,F).

**Figure 4:**
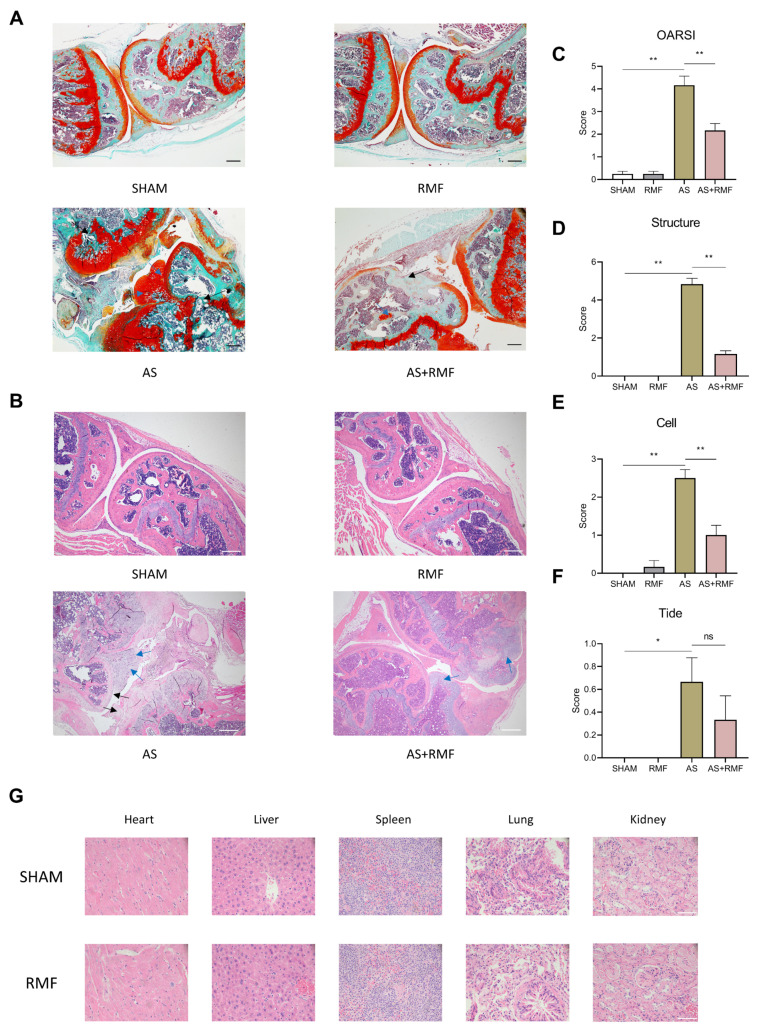
RMF alleviates the articular degradation in AS mice without apparent organ toxicity. (**A**,**B**) Safranin O/Fast green and H&E staining (*n* = 6, scale bar: 200 μm) analysis of murine knee joints. The black arrows display the deteriorated articular cartilage tissue; the blue arrows show ectopic novel cartilage tissue formation in the articular cavity. (**C**–**F**) The OARSI score is utilized to determine the articular deterioration of the cartilage tissue. Data are presented as the mean ± SEM (*n* = 6). * *p* < 0.05, ** *p* < 0.01, ns, no significance, by one-way ANOVA. (**G**) Representative H&E staining images (*n* = 6, scale bar: 50 μm) of the heart, liver, spleen, lung, and kidney of sham and RMF-treated mice.

To further evaluate the positive effect of RMF on AS-like symptoms in CD4-CKO mice, cartilage tissues from the hind legs of 12-month-old mice were stained with safranin O/fast green and H&E (hematoxylin and eosin). Such staining was applied to assess the erosion, hypocellularity, and proteoglycan loss in the superficial articular cartilage. It was discovered that following RMF treatment, the AS mice exhibited more intact cartilage surfaces and denser proteoglycan (Figure 4A,B). RMF depressed OARSI and Mankin scores in AS mice compared to the non-RMF AS group (Figure 4C–F). H&E staining of the heart, liver, spleen, lung, and kidney in the SHAM and RMF groups indicated no significant organic toxicity with RMF treatment (Figure 4G).

### 3.3. RMF Mitigates the Immune Dysfunction of AS Mice with Cartilage Deformation

**Figure 5:**
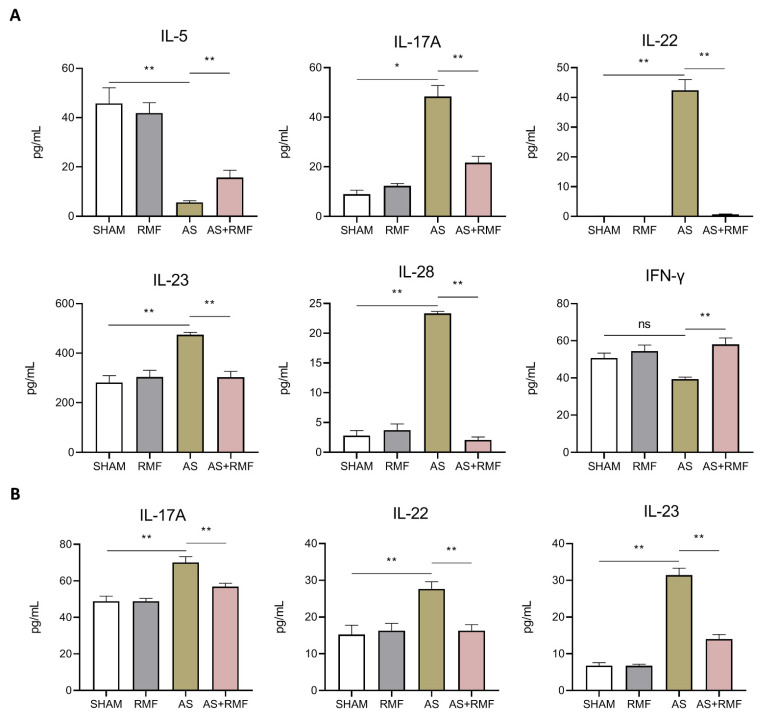
RMF mitigates the immune dysfunction of AS mice. (**A**) Serum levels of IL-5, IFN-γ, IL-17A, IL-22, IL-23, and IL-28 expression (*n* = 6, CBA detection). (**B**) Serum levels of IL-17A, IL-22, and IL-23 expression (*n* = 6, ELISA detection). (**A**,**B**) Data are presented as the mean ± SEM. * *p* < 0.05, ** *p* < 0.01, ns, no significance, by one-way ANOVA.

Serum cytokines were measured with a sensitive and efficient technique known as the cytometric bead assay (CBA). RMF relieved elevated levels of IL-17A, IL-22, IL-23, and IL-28, as well as reduced levels of IL-5 and IFN-γ (Figure 5A). Following this, the levels of IL-17A, IL-22, and IL-23 in murine serum were examined using enzyme-linked immunosorbent assay (ELISA) kits (Figure 5B), and the results were consistent with those obtained using the CBA.

**Figure 6:**
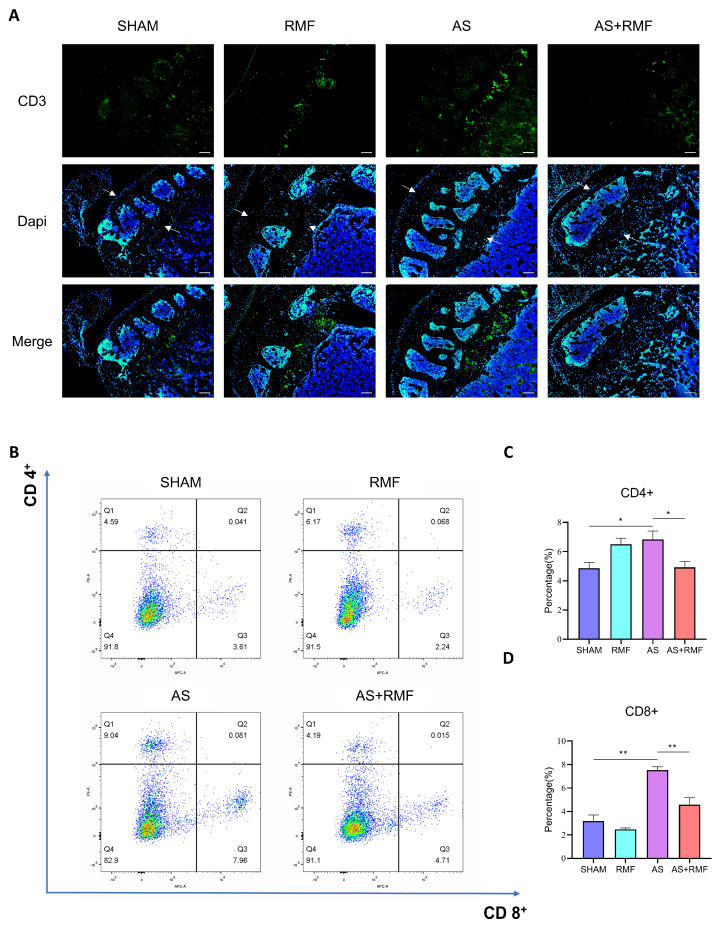
RMF ameliorates the immune malfunction of AS mice with cartilage damage. (**A**) Cartilage tissues from the treated mice at 12 months were stained with an antibody specific for CD3 (*n* = 6, scale bar: 100 μm). White arrows label the cartilage layer on the articular surface and the epiphyseal cartilage layer. (**B**) Flow cytometry detection of the percentages of CD4^+^ and CD8^+^ cells in murine spleens (*n* = 6). (**C**,**D**) The proportion of CD4^+^ and CD8^+^ cells (*n* = 6). (**C**,**D**) Data are presented as the mean ± SEM. * *p* < 0.05, ** *p* < 0.01, by one-way ANOVA.

Immunofluorescence investigations revealed an upsurge of CD3^+^ cells in bone slices from AS mice. However, the positive rate dropped drastically after RMF treatment (Figure 6A). Additionally, the 12-month-old recipient with KO-CD4^+^ T-cells exhibited bone abnormalities with apparent inflammation in the skin, which was partially alleviated by RMF treatment.

Considering that CD4-CKO animals exhibited immunological impairments, it was hypothesized that RMF would adjust T-cell conversion. Afterwards, spleens were extracted from 12-month-old mice, and splenic lymphocytes were isolated and quantified by flow cytometry for the amount of CD4^+^ and CD8^+^ expressing cells. RMF treatment dramatically decreased CD4^+^ and CD8^+^ T-cell subgroups in the spleens of elderly AS + RMF mice compared to the AS group (Figure 6B). Following RMF treatment, the absolute cell counts plummeted, both for the whole amount and for their CD4^+^ and CD8^+^ T-cell subsets, particularly the CD4^+^ T-cells, which were tremendously attenuated in the RMF-treated AS mice (Figure 6C,D).

### 3.4. RMF Treatment Rebalances the Bone Redox System and Energy Metabolism of AS Mice

**Figure 7:**
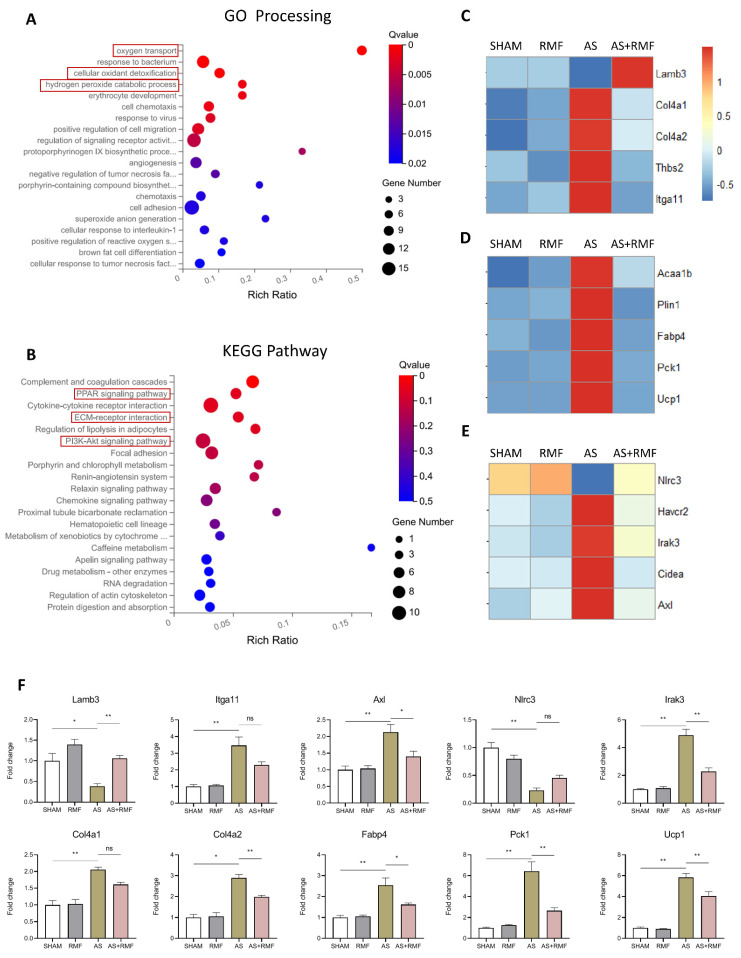
RMF mitigates skeletal disorders by adjusting the AS mice’s bone redox system and energy metabolism. (**A**) Candidate signaling pathways of co-targets between AS and AS + RMF mice based on Gene Ontology (GO) enrichment analysis (*n* = 4). Red frames represent candidate pathways in GO enrichment analysis. (**B**) Candidate signaling pathways of co-targets between AS and AS + RMF mice based on Kyoto Encyclopedia of Genes and Genomes (KEGG) enrichment analysis (*n* = 4). Red frames represent candidate pathways in KEGG enrichment analysis. (**C**) Heatmap of Lamb3, Col4, Thbs2, and Itga11 expression differences in cartilage tissues (*n* = 4). (**D**) Heatmap of Acaa1b, Plin1, Fabp4, Pck1, and Ucp1 expression differences in cartilage tissues (*n* = 4). (**E**) Heatmap of Nlrc3, Havcr2, Irak3, Cidea, and Axl expression differences in cartilage tissues (*n* = 4). (**F**) The expression of Lamb3, Itga11, Axl, Nlrc3, Irak3, Col4a1, Col4a2, Fabp4, Pck1, and Ucp1 was evaluated via qPCR (*n* = 4). Data are presented as the mean ± SEM. * *p* < 0.05, ** *p* < 0.01, ns, no significance, by one-way ANOVA.

To further elucidate the mechanism by which RMF prevents the progression of AS-like changes in mice, murine RNA from cartilage tissue was sequenced using whole-transcriptomic sequencing. By identifying differentially expressed genes between AS and AS + RMF groups according to the GO-processing route, it was found that they were primarily consolidated in the redox and energy metabolism systems (Figure 7A,B). Subsequently, utilizing GO-processing network enrichment analysis, it was exhibited that the differentially expressed genes were predominantly associated with the following pathways: cellular oxygen transport, cellular oxidant detoxification, and hydrogen peroxide catabolic processes (Figure 7A). Additionally, the KEGG pathway enrichment analysis revealed that the differentially expressed genes between the AS and AS + RMF groups were preponderantly aligned with the relevant pathways: ECM-receptor interaction, PI3K-Akt signaling pathway, and PPAR signaling pathway (Figure 7B). For subsequent investigations, the deterioration of the ECM was concentrated on. RNA-seq analysis of cartilage tissue in AS mice treated with RMF exhibited a substantial rise in Lamb3 and a decline in expression of Col4, Thbs2, and Itga11, which are involved in the rebalancing of ECM production (Figure 7C). Additionally, RMF treatment dramatically decreased the expression of lipid metabolism genes in the PPAR signaling pathway, especially Acaa1b, Plin1, Fabp4, Pck1, and Ucp1 (Figure 7D). Interestingly, decreased expressions of Havcr2, Irak3, Cidea, and Axl were noticed in the AS-RMF group (Figure 7E), all of which are implicated in the TNF-α pathway. The validation of qPCR with RNA-seq exhibited an analogous pattern (Figure 7F).

**Figure 8:**
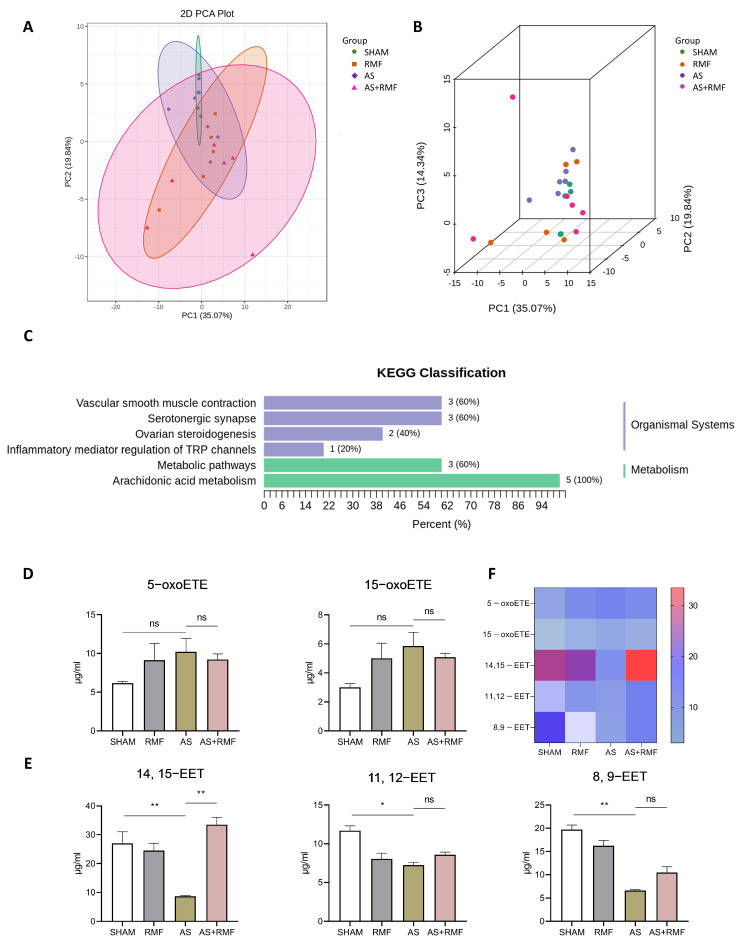
RMF adjusts skeletal lesions by regulating the arachidonic acid metabolism of AS mice. (**A**,**B**) The PCA analysis of serum specimens from four groups of mice (*n* = 4). (**C**) Differential metabolic classifications of the KEGG enrichment map (*n* = 4). (**D**,**E**) The concentrations of 5−oxoETE, 15−oxoETE, 14, 15−EET, 11, 12−EET, and 8, 9−EET in serum specimens from four groups of mice based on metabolomics analysis (*n* = 4). (**F**) Heatmap of 5−oxoETE, 15−oxoETE, 14, 15−EET, 11, 12−EET, and 8, 9−EET in murine serum specimens (*n* = 4). (**D**,**E**) Data are presented as the mean ± SEM. * *p* < 0.05, ** *p* < 0.01, ns, no significance, by one-way ANOVA.

Metabolomics detection was employed to further investigate the impact of RMF on the metabolism of AS mice. Principal component analysis (PCA) was utilized as a dimensionality reduction step in the clustering procedure (Figure 8A,B). The data displayed a partial separation among the four groups, which indicates that the differences among the groups were significantly dispersed, whereas the differences within each group were minute. Furthermore, the KEGG classification (Figure 8C) exhibited modifications in arachidonic acid metabolism, metabolic pathways, inflammatory mediator regulation of TRP channels, ovarian steroidogenesis, serotonergic synapse, and vascular smooth muscle contraction. Combined with the findings mentioned above, the arachidonic acid (ARA) metabolism was emphasized. The concentrations of epoxyeicosatrienoic acid (ETE) and eicosatetraenoic acid (EET) were notably different between the two groups. A considerable decrease in the levels of 5−oxoETE (5−oxo−6E, 8Z, 11Z, and 14Z−ETE) and 15−oxoETE (15−oxo−5Z, 8Z, 11Z, and 13−ETE) was detected in AS mice, whereas RMF treatment substantially reversed this trend (Figure 8D). Additionally, in all four groups, the 14, 15−EET ((±)14(15)−5Z, 8Z, 11Z−EET), 11, 12-EET ((±)11(12)−5Z, 8Z, 14Z−EET), and 8, 9−EET ((±)8 9−5Z, 11Z, 14Z−EET) concentrations drastically decreased, whereas the RMF treatment increased these EET concentrations (Figure 8E). The general alterations of these metabolites were demonstrated in a heatmap (Figure 8F).

### 3.5. RMF Alleviates Bone and Cartilage Deterioration via the RANKL/RANK/OPG Signaling Pathway

**Figure 9:**
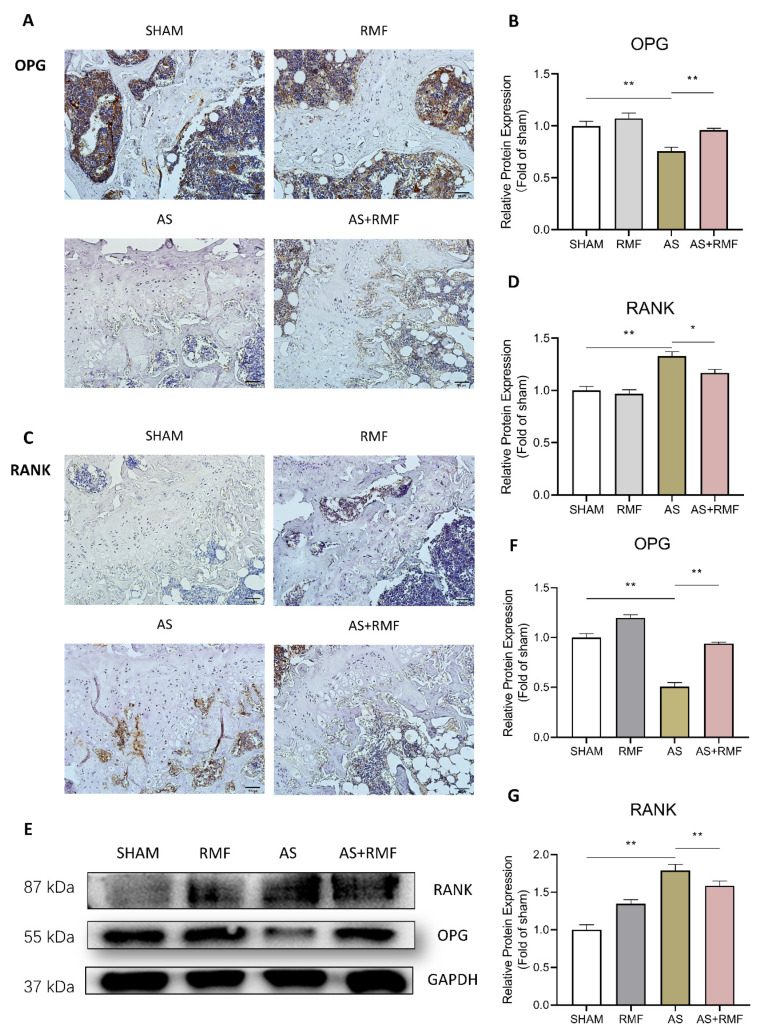
RMF moderates bone and cartilage degradation through the RANKL/RANK/OPG signaling pathways. (**A**,**C**) Immunohistochemistry (IHC) detection of OPG and RANK antibodies in bone slices among four groups of mice (*n* = 4, scale bar: 50 μm). (**B**,**D**) The relative OPG and RANK protein expression in bone and cartilage tissues (*n* = 4). Five regions of interest were randomly selected in each sample. The mean optical density of each region was measured, and the mean value of the five regions’ mean optical density was calculated as the relative positive protein expression. The baseline was the mean optical density of the sham mice, and the relative protein expression in each specimen was calculated as the ratio of the optical density of each tissue versus the baseline. (**E**) Western blot analysis of OPG and RANK protein expression levels in cartilage tissues of four groups of mice (*n* = 4). (**F**,**G**) The relative OPG and RANK protein expression in bone and cartilage tissues (*n* = 4). The gray values of the target proteins were first normalized with the loading control (GAPDH). After calculating the mean gray value of the sham mice as a baseline, the relative protein expression was determined by the ratio of the normalized gray value of each sample to the baseline. (**B**,**D**,**F**,**G**) Data are presented as the mean ± SEM. * *p* < 0.05, ** *p* < 0.01, by one-way ANOVA.

Eventually, in order to validate osteoclast development in the bone canal of AS mice, the femurs were paraffined, sectioned, and stained with RANK- and OPG-specific antibodies. The immunohistochemistry (IHC) analysis indicated that, in comparison to the SHAM group, the AS group exhibited additional fat vesicular cavities in the bone cavity, whereas OPG expression dropped dramatically (Figure 9A,B). OPG expression was elevated in the AS group following RMF treatment, while RANK expression was substantially reduced (Figure 9C,D). Following quantification, it was found that the relative positive fraction of OPG^+^ cells in the bone canal of AS mice declined by approximately 50% compared to the SHAM group, yet this tendency was restored following RMF treatment (Figure 9B). In addition, RANK expression in the bone canal of the mice was contradictory to that of OPG, the expression of which was approximately 150% stronger in AS mice than in SHAM mice (Figure 9D), while RMF treatment reversed this trend (Figure 9C,D). Following that, total protein was isolated from the bone lumen of mice, and Western blotting was utilized to assess the quantities of OPG and RANK expression. The results followed a pattern roughly comparable to that observed in the IHC (Figure 9E–G).

## 4. Discussion

This study aims to demonstrate the efficacy of RMF in AS-like mice generated by CD4-CKO gene editing, which exhibits the formation of ectopic extra bone, bone fusion, and osteoporosis, all of which result in spinal ankylosis and potentially lifelong disability. Specifically, SHP2 was knocked down in proliferative chondrocytes in CD4-CKO-AS mice, leading to a disruption of the growth plate fusion [54,59]. During endochondral ossification in AS, dysfunctional chondrocytes in the growth plate and enthesis triggered the abnormal development of new bone [54,60]. Nevertheless, RMF treatment corrected these pathological alterations by inhibiting inappropriate chondrocyte proliferation and restraining erroneous osteo-epiphysis occlusion in this study. These data indicate that RMF might efficiently delay abnormal new bone growth and radiographic degeneration via targeting chondrocytes in AS mice.

Two paradoxes have been observed in the clinical characteristics of AS: (1) cartilage deterioration is associated with osteoproliferation, and (2) osteoporosis co-exists with bone development in AS [61,62,63]. It has been hypothesized that osteoproliferation in the joints accelerates osteoclast differentiation via interactions between osteoblasts and osteoclasts or between chondrocytes and osteoclasts. Intriguingly, both adult and elderly CD4-CKO animals had degenerated articular cartilage and thicker growth plates in knee segments, demonstrating that deterioration occurs only in the articular cartilage and that osteoproliferation occurs via the growth plate. Chondrogenesis is responsible for the development of malignant new bone in AS ligaments; hence, the inhibition of the osteogenesis process might be a direct mechanism of RMF to halt the radiological progression of AS.

RMF has been seen to impede the proliferation of aberrant cartilage in the articular compartment. Furthermore, it has been confirmed that MF promotes cartilage restoration in patients with osteoarthritis by enhancing chondrocyte proliferation and proteoglycan synthesis, as well as relieving pain and facilitating athletic performance [64]. Correspondingly, recent research using the CD4-CKO-AS mouse model has demonstrated that the cause of cartilage hyperplasia is the malfunction of the narrow growth plate, resulting in disorders of the chondrogenic differentiation inhibitory signal and subsequent hyperplasia [65]. Furthermore, it has been reported that growth plate closure dysfunction and cartilage hyperplasia in CD4-CKO-AS mice are not directly related to immune system dysfunctions [65]. Combined with the findings in this study, these results indicate that RMF contributes to the improvement of articular luminal cartilage defects in AS mice in ways other than alleviating inflammation and promoting osteogenesis.

Additionally, MFs have been illustrated to stimulate the proliferation, synthesis, and secretion of growth factors in bone tissues [66]. It serves as an adenosine A2 and A3 agonist in the chondrocytes of the joints, resulting in anti-inflammatory and chondroprotective actions [66]. In multiple configurations, RMF affects bone metabolism and osteoclast differentiation [49]. It promotes osteoclast differentiation, bone mineral density, and bone resorption [67]. This is also corroborated by the performance of the RMF treatment in this study. Furthermore, there was no substantial difference between the RMF and SHAM groups in any area of the bone and joint cavities after up to 10 months of RMF treatment. Consequently, RMF is predicted to suppress excessive inflammatory responses in bone and joint disorders, raise bone density, and promote osteogenesis and osteoclast differentiation in bone metabolism.

Our imaging data demonstrate that RMF prevented the formation of ectopic osteophytes, scoliosis, and chondrosarcomas in AS mice, as well as interrupted the progressive loss of motor functions in these mice. Combined with X-ray and micro-CT analysis, it was found that the downward trend in BMD and bone loss in AS mice could be prevented by the RMF treatment. As revealed by Safranin O/Fast green staining, RMF treatment relieved malformed cartilaginous hyperplasias and reduced joint deformities in AS mice. Meanwhile, RMF has also been observed to restore the athletic capacity in AS mice. All findings suggest that RMF is effective in relieving AS-like bone deformities.

It was identified that the most probable pathophysiology of AS comprises aberrant chondrogenesis and maturation, as well as inflammatory-mediated osteoclast differentiation [68]. The whole transcriptome analysis revealed that the RMF treatment contributes to the prevention of ECM degradation. Furthermore, AS mice have decreased expression of peroxisome proliferator-activated receptor γ (PPAR-γ), a ligand-activated transcription factor belonging to the nuclear hormone receptor superfamily, which RMF reverses.

Additionally, through metabolomics, we observed metabolic alterations related to lipid oxidation and various arachidonic acid metabolites in these groups. The RMF treatment considerably alleviated the decrement of certain metabolites in the serum of AS mice. Arachidonic acid (ARA), a polyunsaturated fatty acid with an omega−6 chain, is a critical unsaturated fatty acid vital for the body’s natural growth and development. During embryonic development, ARA is an essential fatty acid critical for the maturation of human tissues and organs, particularly the brain and nervous system [69]. Eicosatetraenoic acid (EET) is a byproduct of the cytochrome P450 surface oxidase metabolism of ARA [70]. EET is a polyunsaturated fatty acid discovered in high abundance in the human body and exhibits anti-inflammatory, anti-apoptotic, and antioxidant properties. Several investigations have also demonstrated that EET impairs mitochondrial division [61]. FABP4 (also known as AP2) is a cytoplasmic fatty acid chaperone that inhibits PPAR-γ, hence modulating adipogenesis, cartilage osteogenesis, and adipogenic differentiation [71]. ARA and its analogs act as natural PPAR-γ ligands. The metabolomics of AS mice showed that five ARAs were down-regulated, which was comparable to the transcriptional PPAR-γ, whereas RMF treatment reversed these patterns. As an immediate regulator of C-FOS expression, PPAR-γ is a transcription factor involved in osteoclast development [72]. In the osteoclast cell line, the lack of PPAR-γ impairs osteoclast development via impairing the RANKL signaling pathway, resulting in aberrant osteoclastogenesis [73].

Integrin 11 (Itga11) is a collagen receptor proven to enhance osteogenic development [74]. RMF also modulated high levels of COL4a1 and Itga11 in the AS group, indicating that the anti-inflammatory effects of RMF depend on the osteoclast differentiation mechanism. Through multifunctional analysis, it was affirmed that limited ARA levels, combined with low PPAR-γ expression, contribute to plentiful chondrogenic differentiation of bone marrow progenitor cells, and Itga11 also plays an integral part in chondrogenic differentiation, indicating that the ratio of osteogenesis could be drastically reduced. In addition, multiple analyses confirmed that RMF treatment reversed the bone loss and BMD reduction in AS mice, as well as high levels of IL-17A, IL-22, and IL-28 in murine serum.

Inflammation plays a critical role in the initiation and progression of bone and joint disorders [75]. IL-17A is a multifunctional cytokine generated by T-assisted (Th) 17 cells and is involved in the development of a variety of autoimmune and inflammatory disorders. Multiple studies have demonstrated that IL-17A considerably stimulates the expression of the nuclear factor-B ligand (RANKL) receptor activator and dramatically suppresses the expression of osteoprotegerin (OPG), thus affecting bone metabolism and enhancing inflammatory responses [76]. IL-22, a marker for RA, is abundantly expressed in the synovial fluid of RA patients and cooperates with NF-κB to promote osteoclast development [77]. IFN-γ could suppress IL-22’s pro-osteoclastogenic effect [77], while IL-23 is a critical constituent of IL-22 synthesis [78]. CBA and ELISA observations indicate that osteoclasts and the RANKL/RANK/OPG pathway are critical components of RMF treatment for alleviating bone deterioration and degeneration in mice with AS. As a result, it is hypothesized that RMF treatment impacts the differentiation trend of bone marrow progenitor cells in the joint and bone lumens of AS mice.

Protein levels of RANK and OPG, critical players in bone formation [79], were further investigated. Moreover, it was discovered that RMF dramatically decreased the expression of TNF-α pathway components, including Havcr2, Irak3, Cidea, and Axl, in this study. Subsequent investigations have revealed that the rehabilitation effects of RMF in the treatment of AS are mediated through the conversion balance of osteoblasts and osteoclasts and anti-inflammation effects. Given that several studies have suggested that inflammation-induced osteogenesis malfunction is detrimental to the homeostasis of the bone microenvironment [80], it was hypothesized that RMF alleviates AS via modulating the inflammatory milieu.

Combined with the multi-omics analysis, predictions regarding plausible molecular mechanisms of RMF treatment in AS have been established. The CARD domain 3 (NLRC3) of the Nod-like receptor (NLR) family was one of the genes with decreased expression in the transcriptome sequencing of AS mice in comparison to the SHAM group. NLRC3 is an intracellular member of the NLR family and was originally discovered in T-cells. NLRC3 is expressed at a reduced level in bone marrow cells and acts as an inhibitor of inflammatory signaling [81]. The absence of NLRC3 stimulates the differentiation of CD4^+^ T-cells into Th1 and Th17 subsets, leading to the development of various diseases [82]. NLRC3 is abundantly expressed in T-cells, and a growing amount of evidence indicates that it performs as a suppressor of T-cell response [81,82]. NLRC3 depletion in AS mice might account for the substantial inflammatory response and elevated IL-17A production. The increased expression of NLRC3 in the AS+RMF mice sheds light on why MF impacts the immune system and mitigates the inflammatory process. Interleukin-1 receptor-associated kinase 3 (IRAK3) has been proven to be overexpressed in arthritis and to perform antagonistically in Toll-like receptor (TLR)-mediated cellular signaling [83,84]. In CHON-001 cells, it silenced IRAK3-restricted IL-1β-induced inflammation and apoptosis [85]. Nevertheless, overexpression of IRAK3 partially reverses the effects of Mir-591 on OA chondrocyte viability by boosting apoptosis and inflammation [86]. In addition, this study found that RMF treatment reduced IRAK3 expression in AS mice, suggesting it as a potential biological target for RMF treatment of bone and joint disorders.

With spontaneous inflammation, bone, and joint damage in AS mice, RMF is expected to normalize the immune-skeletal discrepancy. In this investigation, data showed that RMF treatment decreased CD3-expressing proliferative cells via immunostaining and reduced CD4^+^/CD8^+^ T-cells via flow cytometry in AS mice. In summary, RMF has been proposed to alleviate the degradation of cartilage matrix and osteophyte formation in AS mice by improving the inflammatory injury of bone and cartilage, regulating the metabolic balance of arachidonic acid metabolites, and modulating the RANKL/RANK/OPG signaling pathway.

## 5. Conclusions

In this study, RMF was observed to inhibit excessive bone ossification and prevent bone deformation in AS mice. Simultaneously, RMF has been proven to improve bone density, facilitate osteogenesis, and promote the differentiation of osteoclasts. In summary, it has been demonstrated that the RMF treatment mitigates cartilage degradation in AS mice by regulating the immune balance and bone metabolic homeostasis. These results suggest that RMF has the potential to be an effective tool for the treatment and rehabilitation of patients with AS.

## Figures and Tables

**Table 1:** Bone disease severity score.

Score	Description
0	None
1	Enlarged pelvic incidence angle
2	Slight stiffness of knee joints
3	Scoliosis, kyphosis, and moderate stiffness of knee joints
4	Marked scoliosis and kyphosis, ankylosis of knee and hip joints
5	Ankylosis of the lower part of the body and spine

**Table 2:** Athletic ability deterioration score.

Score	Description
1	Normal movement
2	Mild dyskinesia
3	Inferior athletic capacity
4	Severe dyskinesia or motor incapacity

**Table 3:** Fatigue rotating score.

Score	Crawling Time
1	≥5 min
1.5	4.5–5 min
2	4–4.5 min
2.5	3.5–4 min
3	3–3.5 min
3.5	2.5–3 min
4	<2.5 min

**Table 4:** Treadmill score.

Score	Number of Electric Shocks
1	≤2
1.5	3–5
2	6–8
2.5	9–11
3	12–14
3.5	15–17
4	>17

**Table 5:** Primer sequences.

Primer	Sequence
β-actin-F	AATCGTGCGTGACATCAAA
β-actin-R	ATGGATGCCACAGGATTCCATACCC
Lamb3-F	TTCGCTGCCTGACTTGACA
Lamb3-R	AAGCGACGGACTGAACTGT
Itga11-F	CACGGCATTTGGCATTGAA
Itga11-R	GTGCCGTAAACCGTAACTT
Axl-F	GAGCCAACCGTGGAAAGAG
Axl-R	CTCGGTTGGCACCTTTCTC
Nlrc3-F	CAGGCGGAGCCCTTTAGCA
Nlrc3-R	GTCCGCCTCGGGAAATCGT
Irak3-F	AAGACCCACGATGGACGAA
Irak3-R	AGAGGTCCAGGGTCGTTTT
Col4a1-F	GGACAAATCGGACCCACTG
Col4a1-R	CCTGTTTAGCCTGGGTGAC
Col4a2-F	CCTCCAGGACAGGGCTTAC
Col4a2-R	GGAGGTCCTGTCCCGAATG
Fabp4-F	AGTGGGAGTGGGCTTTGCC
Fabp4-R	TCCTGTCGTCTGCGGTGAT
Pck1-F	CAGTCATCATCACCCAAGAG
Pck1-R	ATAGGGCGAGTCTGTCAGTT
Ucp1-F	GTACCGAGCTGTGCGATGT
Ucp1-R	TTCCTCCAAGTTGCTTATGTG

## Data Availability

The raw data from this research are available from the corresponding authors.
